# Taxonomic novelties and phylogenetic insights on Gilliesieae (Allioideae, Amaryllidaceae) from Chile

**DOI:** 10.3897/phytokeys.270.175170

**Published:** 2026-01-29

**Authors:** Nicolás García, Paula Zúñiga-Acevedo, Matías A. González, M. Matías Espinoza, Constanza Soto, Benjamín J. Cisternas-Gallardo

**Affiliations:** 1 Herbario EIF & Laboratorio de Evolución y Sistemática, Facultad de Ciencias Forestales y de la Conservación de la Naturaleza, Universidad de Chile, Av. Santa Rosa 11315, La Pintana, Santiago, Chile Universidad de Chile Santiago Chile; 2 Agrupación por la Conservación de la Biodiversidad Reverdecido, Coltauco, Chile Agrupación por la Conservación de la Biodiversidad Reverdecido Coltauco Chile; 3 Agrupación Taguatagua Nativo, San Vicente de Tagua Tagua, Chile Agrupación Taguatagua Nativo San Vicente de Tagua Tagua Chile; 4 Centro de Estudios Agroecológicos y Botánicos, Rizoma, Valparaíso, Chile Centro de Estudios Agroecológicos y Botánicos, Rizoma Valparaíso Chile

**Keywords:** Alliaceae, Central Chile, endemic flora, IUCN, phylogeny, South America, species description, taxonomy

## Abstract

Tribe Gilliesieae (Allioideae, Amaryllidaceae) comprises bulbous plants characterized by their zygomorphic flowers and a distribution restricted to Chile, Argentina, Peru, and Bolivia. The main objective of this article is to introduce four new species of Gilliesieae recently discovered in central Chile, two belonging to *Gilliesia* (*G. reflexa*, *G. taguataguensis*) and two to *Miersia* (*M. nahuelbutensis*, *M. subandina*). Three of the new species are exclusively distributed in the O’Higgins Region, while the fourth has been recorded in the Biobío and La Araucanía regions. In addition, the new species were placed within the phylogenetic context of the tribe to explore their evolutionary relationships. For this purpose, plastid DNA regions (*rbcL* and *trnL-F*) and the nuclear ribosomal DNA spacer (ITS) were amplified and sequenced, and the resulting sequences were analyzed together with molecular data from previous studies. The phylogenetic results support the inclusion of the new species within *Gilliesia* and *Miersia*, respectively, and support a previously suggested broad circumscription of *Gilliesia*, encompassing *Ancrumia*, *Gethyum*, and *Solaria*. We provide morphological descriptions, a distribution map, field images, illustrations, and conservation assessments for the new species, as well as an updated key to all species of Gilliesieae.

## Introduction

Gilliesieae Baker is a South American tribe in Amaryllidaceae J. St.-Hil. subf. Allioideae Herb. that includes several narrowly endemic and threatened species (Torres-Mellado et al. 2012; García et al. 2022a; Escobar et al. 2023) and is characterized by zygomorphic flowers, a character state that is distinctive from the rest of Allioideae, which typically have actinomorphic flowers (Rudall et al. 2002; Escobar et al. 2020; García et al. 2022b). This tribe is currently composed of seven genera mainly distributed in the southern cone of South America: *Ancrumia* Harv. ex Baker, *Gethyum* Phil., *Gilliesia* Lindl., and *Miersia* Lindl. (including *Speea* Loes.) from central Chile; *Solaria* Phil. from Chile and Argentina; *Trichlora* Baker from Peru; and *Schickendantziella* Speg. from Argentina and Bolivia (Escobar 2012; García et al. 2022a; Escobar et al. 2023).

*Miersia* is endemic to central Chile and composed of five species until recently (Escobar 2012; Escobar et al. 2020). This genus includes bulbous herbs with zygomorphic flowers, perigones formed by six free green-violaceous tepals, sometimes very reduced floral appendages (of staminal and tepaliferous origin), and in most species, a staminal tube formed by the fusion of six fertile stamens (Rudall et al. 2002; Escobar 2012; Cádiz-Véliz 2021; García et al. 2022a). During the last few years, three new species of *Miersia* have been described, and the consideration of the former monotypic genus *Speea* as *Miersia humilis* (Phil.) M.F. Fay & Christenh. has been recommended to comply with the principle of monophyly in this genus (Cádiz-Véliz 2021; García et al. 2022a). On the other hand, *Gilliesia* is composed of five species, mostly from central Chile, except *G. graminea* Lindl., which also occurs in Mendoza, Argentina (Escobar 2012; Escobar et al. 2023). *Gilliesia* is characterized by its strongly zygomorphic flowers, perigones formed by four to six tepals, complex unequal floral appendages, and a staminal tube formed by three fertile stamens and three staminodes (Escobar 2012; Escobar et al. 2023). The paraphyly of *Gilliesia* in relation to *Ancrumia*, *Gethyum*, and *Solaria* has been noted in previous studies (Escobar 2012; Escobar et al. 2020; García et al. 2022a); however, a lack of resolution in the phylogeny has prevented a strong suggestion regarding a broader circumscription of the genus to comply with the monophyly principle.

**Figure 1.**
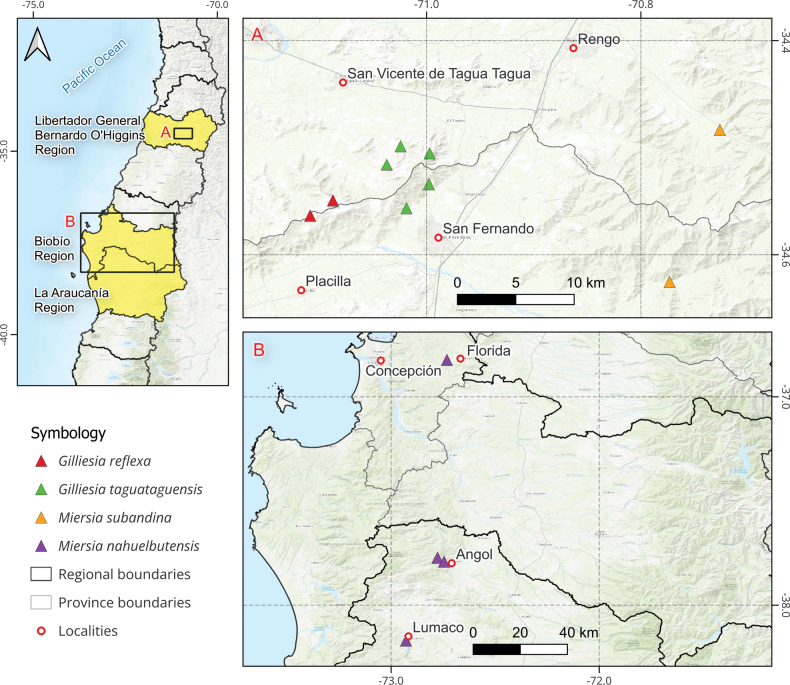
Distribution of new Gilliesieae species. **A**. *Miersia subandina* (orange triangles), *Gilliesia reflexa* (red triangles), and *G. taguataguensis* (green triangles); **B**. *Miersia nahuelbutensis* (purple triangles).

As the result of several independent field explorations in central Chile between 2022 and 2024, four undescribed species of Gilliesieae were discovered (Fig. 1). Three of these new species were found in the O’Higgins Region (34°28'–34° 37'S), while the fourth species was found mainly in the Nahuelbuta coastal mountain range (36°49'–38°10'S). This study describes these new species and provides a distribution map, illustrations, and conservation assessments for them, besides an updated identification key to all species of tribe Gilliesieae. Additionally, the four novel species were placed in the phylogeny of Gilliesieae to evaluate their evolutionary affinities and gain insights regarding generic circumscriptions within the tribe.

## Methods

### Herbarium and fieldwork

Fieldwork to collect the type specimens and silica-dried leaves for DNA extractions was carried out in August 2023 (*Miersia nahuelbutensis*, *Gilliesia reflexa*, *G. taguataguensis*), September 2024, and August 2025 (*Miersia subandina*; Table 1). Specimens were collected and deposited in the collections of the EIF, CONC, and SGO herbaria (Thiers 2025, updated continuously). Additionally, flowers were collected in 70% ethanol for morphological measurements and descriptions. The main taxonomic literature on Gilliesieae was consulted for morphological descriptions of previously described species (Ravenna 2000; Escobar 2012; Escobar et al. 2020, 2023; Cádiz-Véliz 2021; García et al. 2022a). Plant terminology follows Beentje (2012). Measurements were made using a Motic MZ-171 stereomicroscope for structures smaller than 1 cm or with the naked eye for larger structures. All widths were measured over the widest portion of the structure.

**Table 1.** Voucher information and GenBank accession numbers for sequences generated in this study.

Species	Locality	Voucher	ITS	*rbcL*	*trnL-F*
*Gilliesia isopetala*	Santuario de la Naturaleza Cerro Poqui, Coltauco	N. García et al. 6829 (EIF)	PX636081	PX529559	PX529566
*Gilliesia reflexa*	Cerro La Sepultura, San Vicente de Tagua Tagua	N. García et al. 6800 (EIF)	PX636079	PX529557	PX529564
*Gilliesia taguataguensis*	Poza Bruja, San Vicente de Tagua Tagua	N. García et al. 6809 (EIF)	PX636080	PX529558	PX529565
*Miersia nahuelbutensis*	Camino a Capitán Pastene, Lumaco	N. García 6792 (EIF)	PX636075	PX529553	PX529560
*Miersia nahuelbutensis*	Camino a Vegas Blancas, Angol	N. García 6794 (EIF)	PX636076	PX529554	PX529561
*Miersia subandina*	Agua Buena, San Fernando	B.J. Cisternas et al. 40 (EIF)	PX636077	PX529555	PX529562
*Miersia subandina*	Las Nieves, Rengo	B.J. Cisternas et al. 41 (EIF)	PX636078	PX529556	PX529563

### Taxon sampling and phylogenetic analyses

Genomic DNA was extracted from silica-dried leaf material of the four novel species and *Gilliesia isopetala* Ravenna (Table 1) using a modified 2× CTAB method (Doyle and Doyle 1987; Cullings 1992; García et al. 2014). Based on previous studies and sequences available for Gilliesieae (Escobar et al. 2020; García et al. 2022a), we amplified the *rbcL* gene and *trnL-F* intron and spacer, which together form our chloroplast DNA (cpDNA) matrix, and the nuclear ribosomal DNA internal transcribed spacer (nrITS). The amplification of DNA fragments followed the protocols described by Escobar et al. (2020) with the following modifications. For *rbcL*, amplification and sequencing were performed only with primers 1F and 1352, and the PCR protocol included 35 cycles and an extension time of 1.5 minutes. All PCR reactions were done using 12.5 µl of PCR Master Mix SapphireAmp Fast (Takara Bio, Shiga, Japan), 4 µl of nuclease-free water, 2.5 µl of each primer (10 µM), 2.5 µl of BSA at 1 mg/mL, and 2 µl of DNA. Sequencing was performed using the same amplification primers by Macrogen, Chile. We generated 21 new sequences and deposited them in GenBank (Table 1); the remaining sequences were obtained from datasets by Escobar et al. (2020) and García et al. (2022a: table SS1; https://phytokeys.pensoft.net/article/87842/element/5/33/).

Taxon sampling and sequence selection follow García et al. (2022a), including 27 ingroup (Gilliesieae) and six outgroup accessions (Leucocoryneae: 5; Tulbaghieae: 1). Editing and assembling sequences were performed in Geneious Prime 2025.1.3 (https://www.geneious.com). Sequences were aligned using MAFFT v.1.4.0 (Katoh and Standley 2013). A maximum likelihood (ML) analysis was performed for the concatenated matrix of the three loci using RAxML-NG v.1.1.0 (Kozlov et al. 2019), GTR+Γ as the model of molecular evolution, and conducting 100 tree searches using 50 random and 50 parsimony-based starting trees to pick the best-scoring topology. Each locus was considered a separate partition. Subsequently, likelihood bootstrap analyses (Felsenstein 1985) were conducted with the “autoMRE” bootstrap convergence test (Pattengale et al. 2009) and a cutoff value of 0.03, which reached convergence after 450 pseudoreplicates. The ML tree was rooted using *Tulbaghia capensis* L. (Escobar et al. 2020; García et al. 2022a). The concatenated alignment and RAxML output files, as well as maximum likelihood and bootstrap trees including outgroup taxa, are available in Zenodo (doi: 10.5281/zenodo.17372175).

### Conservation assessment

The assessment of the conservation status of both species was conducted using the International Union for Conservation of Nature (IUCN 2024) criteria. The extent of occurrence (EOO) was calculated for species with more than two records (*Miersia nahuelbutensis*, *Gilliesia taguataguensis*) using GeoCat (Bachman et al. 2011). The area of occupancy (AOO) was estimated by tracing polygons of specific habitats (i.e., rocky outcrops, forest fragments) in Google Earth for both species (*Miersia subandina*, *Gilliesia reflexa*) with only two records and with the IUCN default cell width of 2 km in GeoCat (Bachman et al. 2011) for *Miersia nahuelbutensis* and *Gilliesia taguataguensis*. Threats were identified from field observations and literature. Field data on population abundance and trends are unavailable for the new species.

## Results

### Phylogenetic analyses

**Figure 2.**
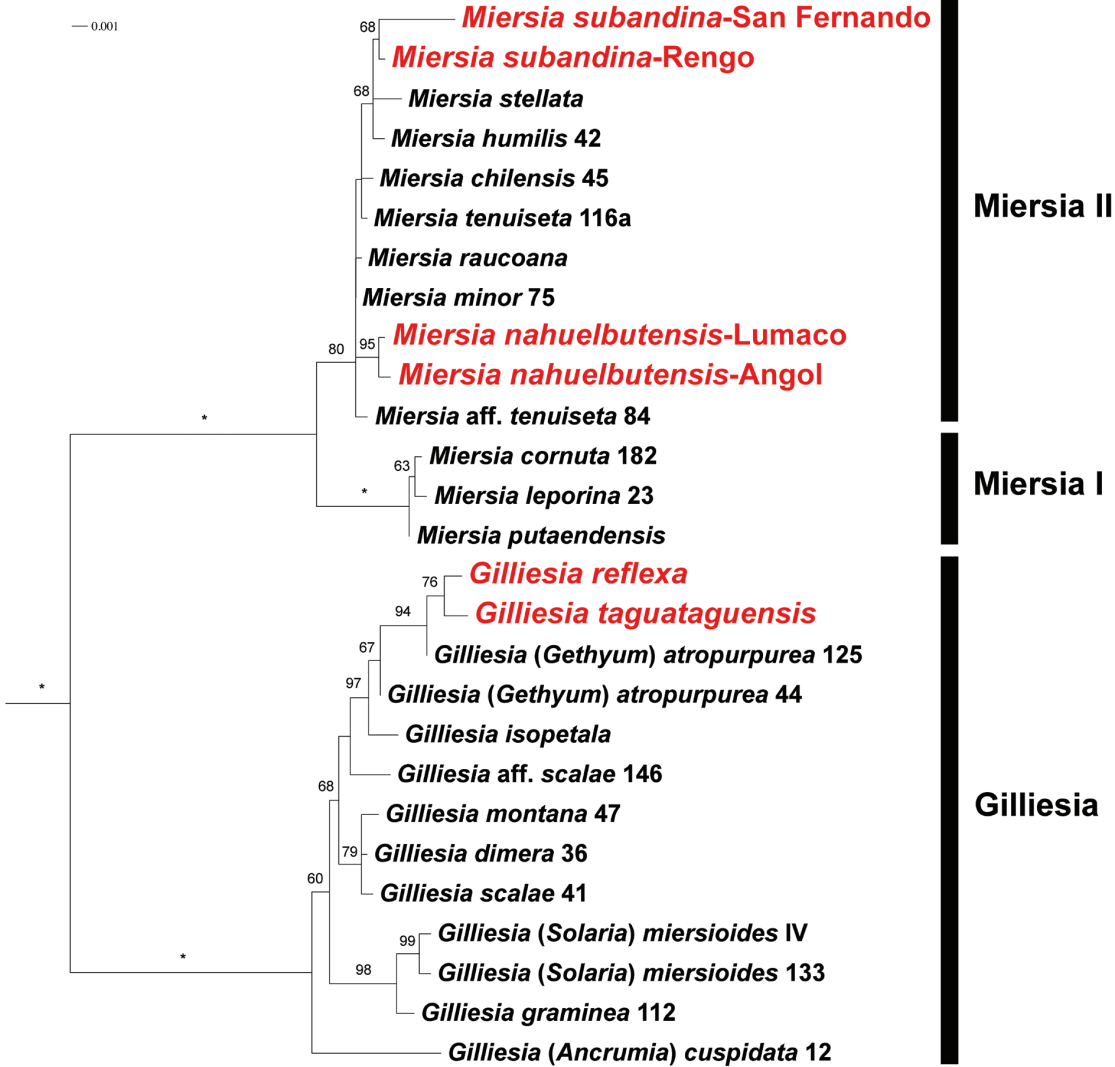
Maximum likelihood phylogram of Gilliesieae based on concatenated analysis of nrDNA ITS and cpDNA (*trnL-F*, *rbcL*). Numbers above branches represent bootstrap (BS) values > 50; asterisks indicate BS = 100. Numbers following species names correspond to accession numbers in Escobar et al. (2020). Novel species placed within the phylogeny are in red font. Names to the right of black bars correspond to informal clade names. Outgroups have been excluded from the figure, and the root branch is not to scale.

Our ML tree overall agrees with the topology reported in previous studies (Escobar et al. 2020; García et al. 2022a) but shows higher support for, and improved resolution within, two major clades: (1) *Gilliesia* s.l., including *Ancrumia*, *Gethyum*, and *Solaria* (BS = 100), and (2) *Miersia* s.l., including *Speea* (BS = 100; Fig. 2). Both *Miersia nahuelbutensis* and *M. subandina* are inferred as members of the *Miersia* II clade (BS = 80); however, this clade includes very short branches and low resolution between species (Fig. 2). On the other hand, *Gilliesia taguataguensis* and *G. reflexa* are retrieved as sister species (BS = 76) within a clade that also includes *Gilliesia atropurpurea* and *G. isopetala* (BS = 97; Fig. 2).

### Taxonomic treatment

#### Miersia nahuelbutensis

Taxon classificationPlantaeAsparagalesAmaryllidaceae

Nic.García sp. nov.

C48DE527-22C7-5A31-A760-1ACC0791BD1A

urn:lsid:ipni.org:names:77375740-1

Figs 3, 4

##### Diagnosis.

*Miersia nahuelbutensis* differs from *Miersia chilensis* Lindl. by its nodding flowers, us/ually with upper tepals and stamens pointing downwards (vs. pointing to the front); upper and lower tepals closer, forming a right angle (vs. tepals wide open); and its longer tepals, 19–20 mm long (vs. shorter tepals, 5–15 mm long).

**Figure 3.**
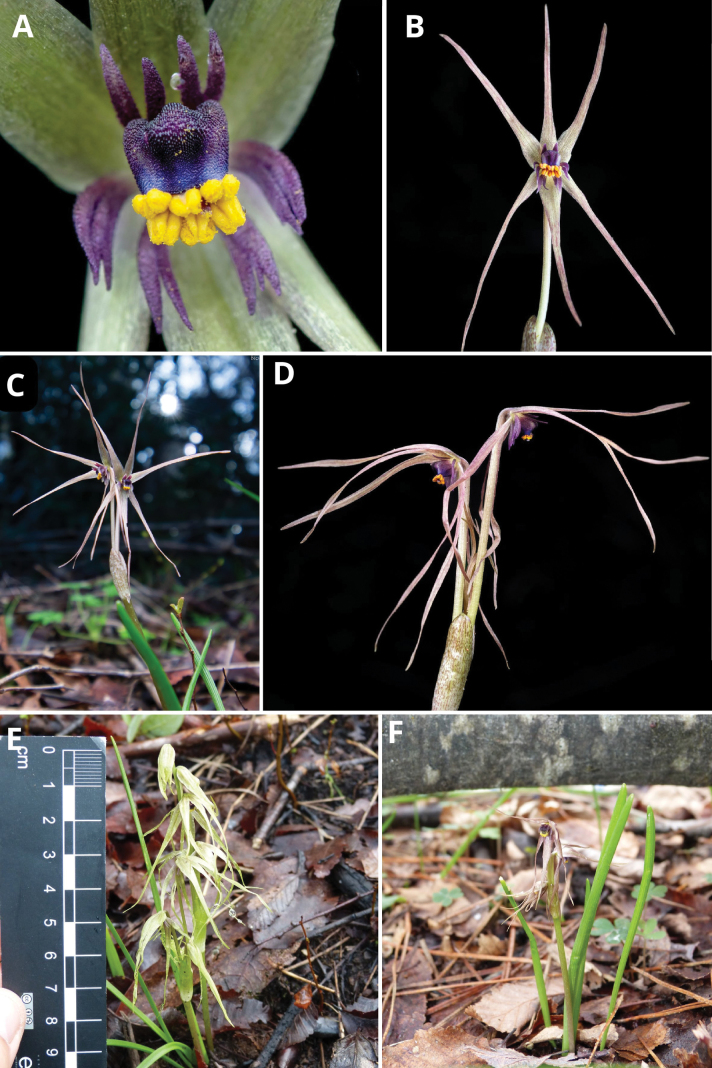
*Miersia nahuelbutensis* Nic. García. **A**. Detail of floral appendages and staminal tube; **B**. Frontal view of flower; **C**. Inflorescence; **D**. Lateral view of flowers; **E**. Habit (Lumaco); **F**. Habit (Angol). Photos by Vicente Valdés (**A**, **B**, **C**, **D**) and Nicolás García (**E**, **F**).

**Figure 4.**
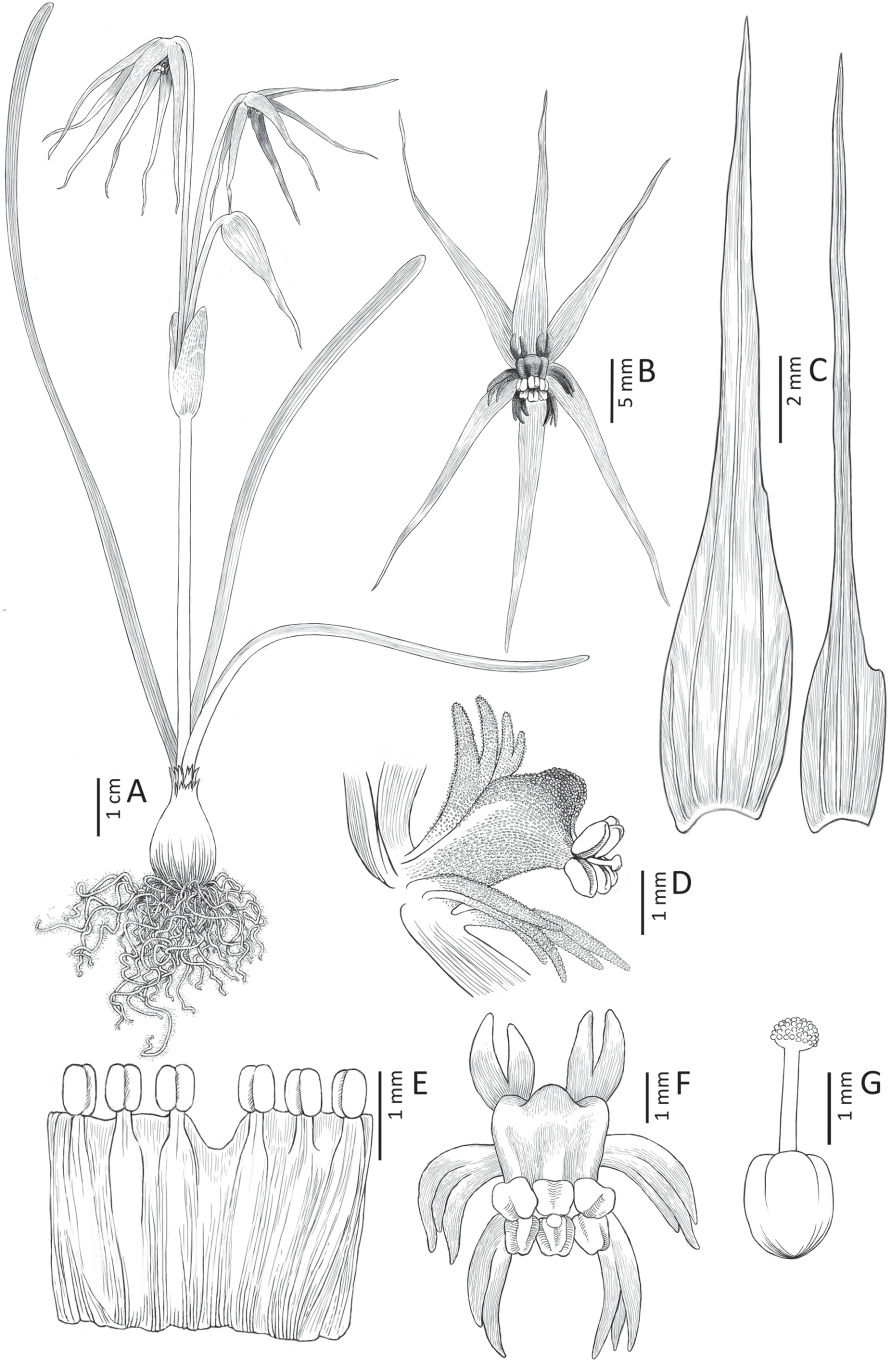
*Miersia nahuelbutensis* Nic. García. **A**. Habit; **B**. Flower (frontal view); **C**. Tepals; **D**. Staminal tube (lateral view); **E**. Staminal tube (open, internal view); **F**. Staminal tube and floral appendages (frontal view); **G**. Gynoecium. Illustration by Daniel Martínez Piña.

##### Type.

Chile • Región de La Araucanía: Provincia de Malleco, Comuna de Angol, camino a Vegas Blancas, 801 m a.s.l., 11 August 2023, *N. García 6794* (holotype: EIF 18603; isotypes: CONC, SGO).

##### Description.

Terrestrial herbs. **Bulbs** subglobose to ovoid, external cataphylls light brown, (11–) 15–25 (–33) × 7–17 mm. **Leaves** 2–3 (–4), linear, 10–16 (–28) × 0.1–0.4 cm. **Scapes** 1 (–3), cylindrical, hollow, (57–) 70–115 (–130) × 0.6–1.5 (–2.0) mm. **Spathe** 2-valvate, herbaceous, lanceolate, 10–16 × 4–5 mm, fused on their basal ½ (~ 4–6 mm), whitish to greenish with 8–11 veins inconspicuous or purple spotted. **Inflorescence** a pseudo-umbel with 2–4 strongly zygomorphic flowers; upper tepals usually forming 90° angle with respect to lower tepals, flowers usually nodding with stamens pointing downwards; **pedicels** unequal, 1.5–3.0 (-4) cm long in open flowers, apex curved in a right angle (~ 90°). **Tepals** six, free, membranous, light green to purplish, lanceolate, caudate, straight, **outer** 19–20 × 1.4–1.6 mm, 3–5 acrodromous veins; **inner** 19–20 × 1.0–1.1 mm, three acrodromous veins; on both whorls only the central is well marked and runs throughout the complete length; cauda 0.4–0.5 mm wide, comprising ~ ¹/5–²/5 of the tepal’s length (4–8 mm). **Floral appendages** purplish, flat, **lateral (tepaliferous)** four, bifid, rarely trifid, one pair on each side, with linear to linear-lanceolate segments, distal half curved downwards, attached to the base of inner tepals, 2.5–3.0 × 0.4–0.8 mm, each segment ~ 1.0–2.0 mm long; **upper (staminal)** two, bifid, rarely entire, with lanceolate to short unequal segments, attached to the base of the staminal tube, 2.5–3.5 (–4) × 0.4–0.5 mm. **Stamens** six, filaments 0.4–0.5 mm long, adnate internally to the staminal tube; **staminal tube** urceolate, purplish with three longitudinal folds on its upper side, single longitudinal fold on the lower side, apex entire, papillose, 2.5–3.0 × 1.2–2.0 mm; **anthers** yellow (purple when dry), 0.5 mm long, exerted. **Ovary** superior, spherical to obovoid, 1.5 mm long, trilocular, 12 ovules per locule, biseriate; **style** straight, exerted, 1.3–2.0 mm long; **stigma** capitate, terminal. **Capsules** obovoid to spherical, 3-valved, 12 × 11–14 mm. **Seeds** (immature) obovoid, 2.3 × 1.5 mm, testa coppery, surface vesicular.

##### Distribution and habitat.

*Miersia nahuelbutensis* was initially recorded in two localities along the eastern side of the Nahuelbuta coastal mountain range in the Malleco Province (Fig. 1B). In both locations it grows in the understory of second-growth forests dominated by *Nothofagus obliqua* (Mirb.) Oerst. (Nothofagaceae), on south- to southwest-facing slopes between 100 and 800 m a.s.l. A codominant species shared by both stands is *Persea lingue* (Ruiz & Pav.) Nees (Lauraceae); other common co-occurring tree species include *Peumus boldus* Molina (Monimiaceae) and *Cryptocarya alba* (Molina) Looser (Lauraceae) in Lumaco, *Gevuina avellana* Molina and *Lomatia hirsuta* (Lam.) Diels (both Proteaceae) in Angol. During the final writing stage of this manuscript, *M. nahuelbutensis* was recorded 100 km north of Angol, close to Florida in the Province of Concepción, Biobío Region, approximately 40 km north of the Biobío River (Fig. 1B), also in a shady forest fragment dominated by *Nothofagus obliqua*.

##### Phenology.

This species has been seen flowering between July and August. Fruits have been recorded during October.

##### Etymology.

The specific epithet refers to the coastal mountain range known as *Cordillera de Nahuelbuta*, where this species was first recorded.

##### Vernacular name.

Although no popular common name is known for *Miersia nahuelbutensis*, we propose to name it “*hadita de Nahuelbuta*,” which means Nahuelbuta’s little fairy.

##### Conservation status.

*Miersia nahuelbutensis* can be considered Endangered (EN) according to criteria B1ab(iii). It has been recorded in four locations, with an estimated EOO of 836.6 km^2^ (< 5,000 km^2^) and AOO of 16 km^2^. In addition, it inhabits a forest type (i.e., P47. Bosque caducifolio mediterráneo interior de *Nothofagus obliqua* – *Cryptocarya alba*) that has been replaced at least in 79.9% of its original area by agriculture and forestry plantations of *Pinus radiata* D. Don and *Eucalyptus globulus* Labill. that is considered a Critically Endangered vegetation belt in Chile (Luebert and Pliscoff 2017).

##### Additional specimens examined (paratypes).

**Chile** • **Región de La Araucanía**: Provincia de Malleco, Comuna de Angol, camino a Vegas Blancas, 801 m a.s.l., 22 October 2018, *N. García 5295* (EIF 15159); • Parque Junquillar, 304 m a.s.l., 16 August 2025, *A. Cádiz-Véliz 1370* (CONC, EIF); • Comuna de Lumaco, camino a Capitán Pastene, 119 m a.s.l., 11 August 2023, *N. García 6792* (EIF, CONC, SGO).

##### Unvouchered observations.

**Chile. Región del Biobío**: Provincia de Concepción, Comuna de Florida, Parque Coyanmahuida, 240 m a.s.l., 23 August 2025, observation by Mauricio Aguirre-Díaz (https://inaturalist.mma.gob.cl/observations/308565645).

#### Miersia subandina

Taxon classificationPlantaeAsparagalesAmaryllidaceae

P.Zúñiga & Nic.García sp. nov.

17414082-16F9-5F87-9F2E-48E02C20D242

urn:lsid:ipni.org:names:77375741-1

Figs 5, 6

##### Diagnosis.

*Miersia subandina* differs from *Miersia humilis* (Phil.) M.F.Fay & Christenh. by a capitate stigma (vs. trilobed stigma), six awl-shaped, rarely flat and bifid, floral appendages (vs. floral appendages absent), and a conical staminal tube (vs. staminal filaments fused in their basal half and covering the ovary, but not forming a conical or urceolate tube).

**Figure 5.**
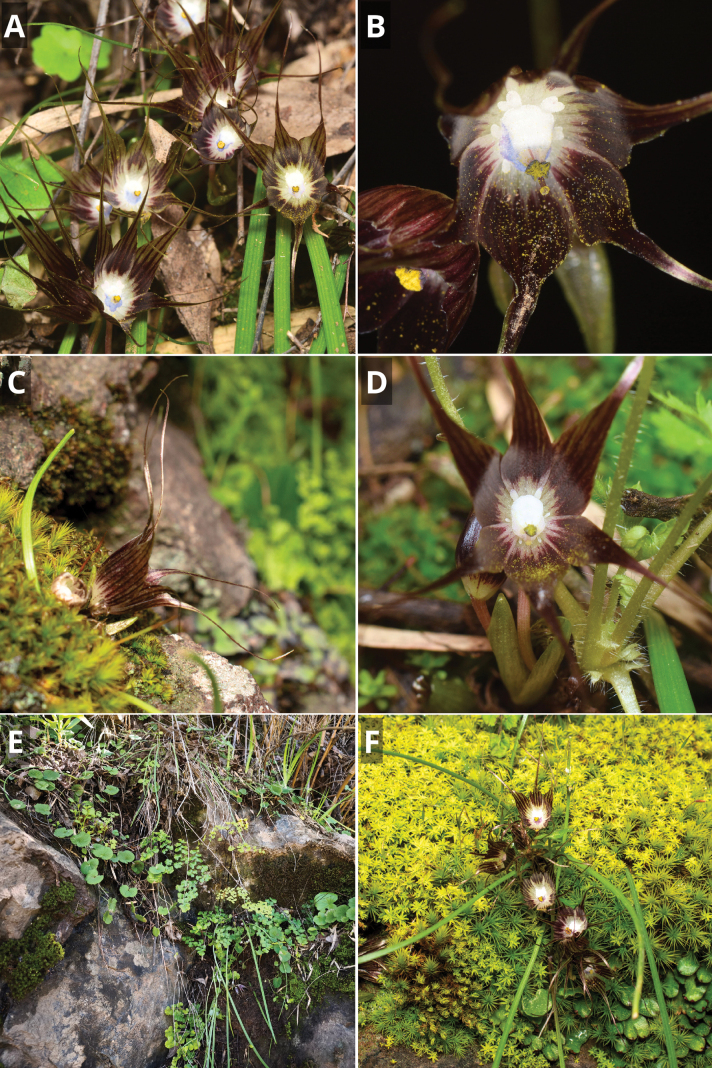
*Miersia subandina* P. Zuñiga & Nic. García. **A**. Flowers; **B**. Detail of staminal tube surrounded by bifid appendages; **C**. Lateral view of flower; **D**. Frontal view of flower; **E**. Habitat; **F**. Habit. Photos by Matías González (**A**, **B**, **E**) and Constanza Soto (**C**, **D**, **F**).

**Figure 6.**
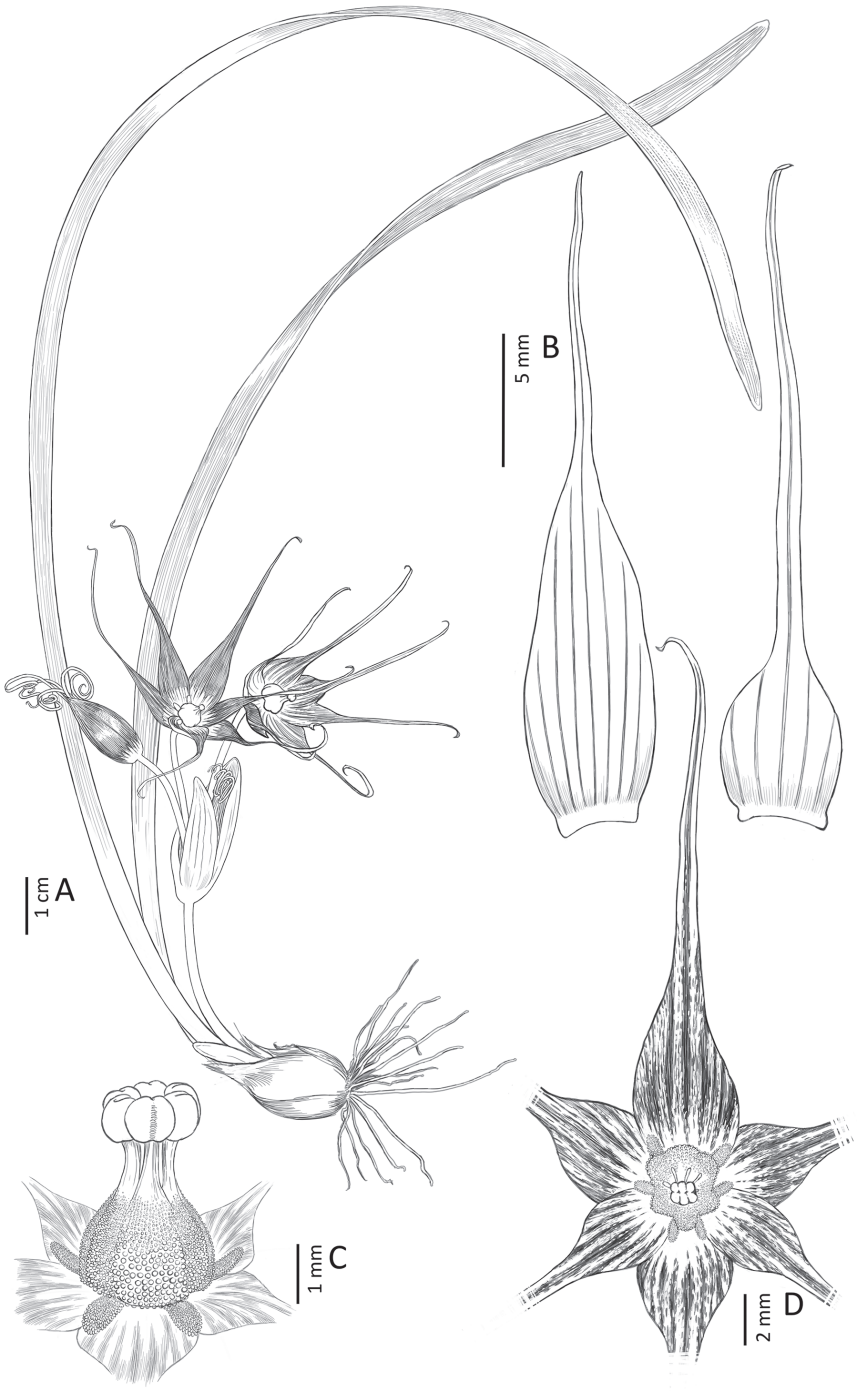
*Miersia subandina* P. Zuñiga & Nic. García. **A**. Habit; **B**. Tepals; **C**. Staminal tube and floral appendages (lateral view); **D**. Flower (frontal view). Illustration by Daniel Martínez Piña.

##### Type.

Chile • Región del General Libertador Bernardo O’Higgins: Provincia de Colchagua, Comuna de San Fernando, estero Antivero, sector Los Alpes, 713 m a.s.l., 26 August 2025, *N. García, P. Zuñiga, L. Santilli, M.A. González, M.M. Espinoza & N. Cáceres 7696* (holotype: EIF 18604; isotypes: CONC, SGO).

##### Description.

Terrestrial saxicolous herbs. **Bulbs** ovoid, external cataphylls light brown, 13–25 × 5–14 mm. **Leaves** 2–3, linear, 12–24 × 0.1–0.3 cm. **Scapes** 1–2, cylindrical, hollow, 35–80 × 0.6–2.0 mm. **Spathe** 2-valvate, herbaceous, lanceolate, 9–16 × 3–4 mm, fused on their basal ½ (~ 4 mm), whitish to greenish with 4–8 veins inconspicuous or purple spotted. **Inflorescence** a pseudo-umbel with 2–4 zygomorphic flowers; **pedicels** unequal, 1.0–1.8 cm long in open flowers, apex straight to slightly curved. **Tepals** six, free, membranous, purplish with whitish base, lanceolate, caudate, straight, **outer** 21–25 (–28) × 4–6 mm, 7–9 acrodromous veins; **inner** 22–25 × 3–4 mm, five acrodromous veins; on both whorls only the central vein is well marked and runs throughout the complete length; cauda 0.4–0.5 mm wide, comprising ~ ½–²/3 of the tepal’s length (12–15 mm); tips curled, especially in bud. **Floral appendages** whitish, usually simple, rarely bifid, awl-shaped, **lateral (tepaliferous)** four, one pair on each side, attached to the base of inner tepals, 0.2–0.7 mm; **upper (staminal)** two, attached to the base of the staminal tube, 0.5–1.1 mm long, tip sometimes purplish. **Stamens** six, filaments 0.7–1.1 mm long, triangular base, born in apical to subapical position from the staminal tube; **staminal tube** conical, sometimes with six longitudinal rounded folds, whitish throughout or with purplish lower half, apex erose and contiguous to filaments, papillose, 1.5–2.0 × 2.5–3.0 mm; **anthers** yellow (purple when dry), 0.3 mm long. **Ovary** superior, obovoid to oblong, 0.8–1.2 mm long, trilocular, 7–10 ovules per locule, biseriate; **style** straight, exerted, 0.7–1.4 mm long; **stigma** capitate, terminal. **Capsules** obovoid to spherical, 3-valved, 4–6 mm long. **Seeds** obovoid, beaked, 2.0–2.5 × 1.5–2.0 mm, testa brownish, surface vesicular to densely foveate.

##### Distribution and habitat.

*Miersia subandina* inhabits the foothills of the main Andes mountains in the Libertador General Bernardo O’Higgins Region. To date, it has been recorded in two localities of the San Fernando and Rengo municipalities, close to the Antivero and Claro rivers, respectively (Fig. 1A). In both locations it grows within shady, south-oriented slopes covered by sclerophyllous forest, in rocky outcrops between 650 and 750 m a.s.l. The main woody species in both locations are *Peumus boldus* (Monimiaceae), *Cryptocarya alba* (Lauraceae), and *Sophora macrocarpa* Sm. (Fabaceae).

##### Phenology.

This species has been recorded in flower between August and September. Fruits have been found during September.

##### Etymology.

The specific epithet refers to the low foothills of the main Andes mountain range, which correspond to the geographical position where this species has been recorded to date.

##### Vernacular name.

Although no popular common name is known for *Miersia subandina*, we propose to name it “*Estrella de los Andes*,” which means Star of the Andes. The name evokes its flowers resembling a star and refers to the fact that this is the only *Miersia* species known to be restricted to the principal Andes mountain range and not found in the Coastal range and/or closer to the sea.

##### Conservation status.

*Miersia subandina* can be considered Endangered (EN) according to criteria B2ab(iii), because its area of occupancy AOO is < 500 km^2^, with an estimate of 6,540 m^2^ (~ 0.007 km^2^) considering both populations. Perturbations of the known populations have been observed due to road maintenance and cattle transiting across the steep slopes it inhabits. In addition, central Chile in general is at risk of forest fires.

##### Additional specimens examined (paratypes).

**Chile** • **Región del General Libertador Bernardo O’Higgins**: Provincia de Cachapoal, Comuna de Rengo, Las Nieves, 622 m a.s.l., 2 September 2024, *B.J. Cisternas, M.A. Gonzalez & M. Contreras 41* (EIF); • Provincia de Colchagua, Comuna de San Fernando, sector Los Alpes, Agua Buena, 700 m a.s.l., 10 August 2024, *P. Zuñiga, C. Soto, M.A. Gonzalez & B.J. Cisternas 39* (EIF); • 2 September 2024, *B.J. Cisternas, M.A. Gonzalez & M. Contreras 40* (EIF).

#### Gilliesia reflexa

Taxon classificationPlantaeEphemeropteraLeptophlebiidae

M.A.Gonz. & Nic.García sp. nov.

7FF325D5-D9E2-5E3C-8B60-E50899C46D00

urn:lsid:ipni.org:names:77375742-1

Figs 7, 8

##### Diagnosis.

*Gilliesia reflexa* differs from *Gilliesia atropurpurea* (Phil.) M.F. Fay & Christenh. by its shorter tepals, 6–10 mm long (vs. (9–)12–25 mm long), and tepals usually strongly reflexed from the base (vs. usually straight or reflexed at tips, sometimes inner tepals reflexed from base).

**Figure 7.**
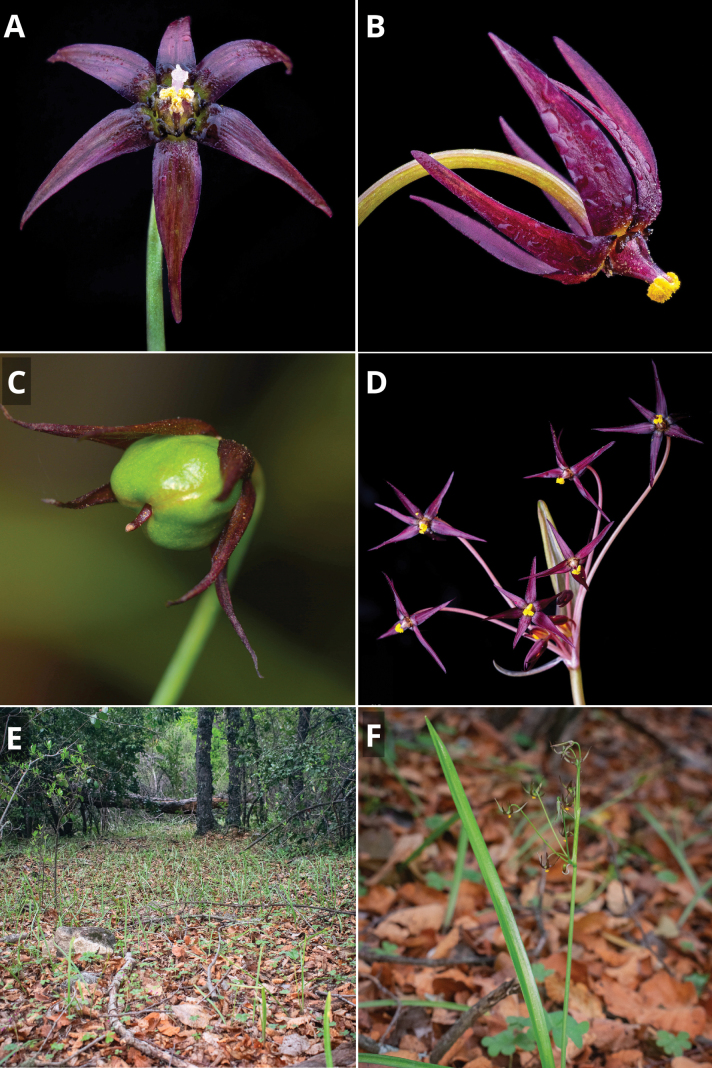
*Gilliesia reflexa* M.A. Gonz. & Nic. García. **A**. Frontal view of flower; **B**. Lateral view of flower; **C**. Immature fruit and persistent tepals; **D**. Inflorescence; **E**. Habitat; **F**. Habit. Photos by Vicente Valdés (**A**, **B**, **D**), Matías González (**C**), and Christofer Olea (**E**, **F**).

**Figure 8.**
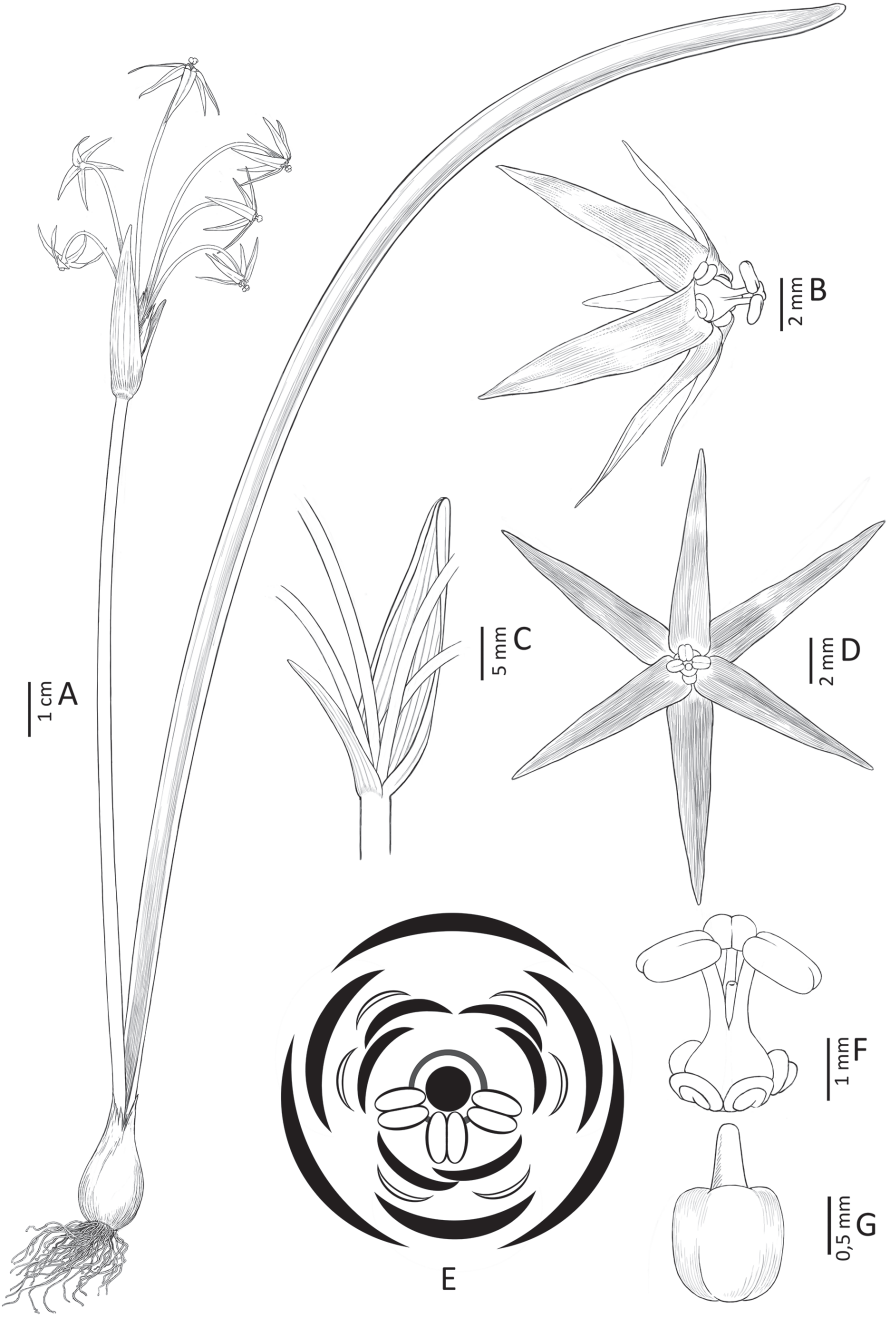
*Gilliesia reflexa* M.A. Gonz. & Nic. García. **A**. Habit; **B**. Flower (lateral view); **C**. Spathe valves and pedicels; **D**. Flower (frontal view); **E**. Floral diagram (large black crescents: tepals; small white crescents: outer appendages; small black crescents: inner appendages; double ellipses: anthers; dark gray line: staminal tube; black circle: gynoecium); **F**. Staminal tube (lateral view); **G**. Gynoecium. Illustration by Daniel Martínez Piña.

##### Type.

Chile • Región del Libertador General Bernardo O’Higgins: Provincia de Cachapoal, Comuna de San Vicente de Tagua Tagua, cerro La Sepultura, Roblería de Rinconada, 1150 m a.s.l., 18 August 2023, *N. García, C.B. Ulloa, M.M. Espinoza. M.A. González, J.A. Hernández & R.A. Alarcón 6800* (holotype: EIF 18605; isotypes: CONC, SGO).

##### Description.

Terrestrial herbs. **Bulbs** ovoid to narrowly ovoid, 21–35 × 7–15 mm, external cataphylls light brown, veins prominent. **Leaves** one, lorate, 32–55 × 0.6–1.0 (–1.3) cm. **Scapes** one, cylindrical, hollow, 19–35 × 0.1–0.3 cm. **Spathe** 2-valvate, herbaceous, green, veins inconspicuous, free, **outer valve** lanceolate, 21–32 × 4–7 mm, veins 11–15, **inner valve** linear, rarely shortly lanceolate, (7–) 10–19 × 1–1.5 (–2.5) mm, veins (1–) 3–5. **Inflorescence** a pseudo-umbel with 6–11 slightly zygomorphic flowers; **pedicels** unequal, 1.8–4.2 cm long (with open flowers). **Tepals** six, free, membranous, vinaceous (purplish red), lanceolate, acute to acuminate, slightly to strongly reflexed from basal third, base papillose, **outer** 7–10 × 1.4–2.0 mm, three inconspicuous veins, **inner** 6.5–10 × 1.2–1.5 mm, one inconspicuous vein. **Floral appendages** vinaceous to burgundy, on two whorls, **outer (tepaliferous)** 2–6, awl-shaped, falcate or obovate with truncate to bifid apex, opposite to inner appendages, attached to the base of inner tepals, 0.1–0.5 mm long, **inner (staminal)** six, nearly orbicular, cordate to semi-circular, arranged in three pairs, each pair with adjacent sides imbricate, 0.4–0.7 mm long. **Stamens** three, filaments 1.0–1.5 mm long, base triangular; **staminal tube** open, contiguous to filaments, vinaceous, papillose, 0.5–0.8 mm long on side with filaments, shorter (0.2–0.4 mm long) or null and exposing gynoecium on side lacking filaments, sometimes with a triangular appendage (staminode); **anthers** three, yellow, dorsifixed near base, bent > 90° with apertures pointing outwards (extrorse), 0.9–1.4 mm long. **Ovary** superior, spherical, 0.7–1.0 mm long, trilocular, 4–5 ovules per locule, biseriate; **style** straight to ascending, 1.1–1.4 mm long, reaching the anthers or exerted in mature flowers; **stigma** capitate, terminal. **Capsules** spherical, 3-valved, dimensions unknown. **Seeds** not seen.

##### Distribution and habitat.

*Gilliesia reflexa* has been recorded only at the top of La Sepultura hill (Fig. 1A), which is one of the tallest peaks in the coastal mountain range that lies between the Cachapoal and Tinguiririca rivers (~ 34.3°S). It has been observed in the understory of two small fragments of relictual forest dominated by *Nothofagus obliqua*, along with other tree species such as *Nothofagus glauca* (Phil.) Krasser (Nothofagaceae), *Quillaja saponaria* Molina (Quillajaceae), *Azara petiolaris* (D.Don) I.M.Johnst. (Salicaceae), and *Aristotelia chilensis* (Molina) Stuntz (Elaeocarpacaea). Both forest fragments are located on south- to southeast-facing slopes at 1050–1150 m a.s.l, with surfaces ranging between four and seven hectares each.

##### Phenology.

*Gilliesia reflexa* has been recorded in flowers from August to September. The fruiting period has been recorded during October.

##### Etymology.

The specific epithet refers to its characteristic reflexed tepals.

##### Vernacular name.

Although no popular common name is known for *Gilliesia reflexa*, we propose to name it “*cometa de La Sepultura*,” which means La Sepultura Comet, due to the resemblance of its flowers to a falling celestial object.

##### Conservation status.

*Gilliesia reflexa* can be considered Critically Endangered (CR) under criteria B2ab(iii), because its area of occupancy is < 10 km^2^, with an estimated 0.1 km^2^ considering both known forest fragments with presence of this species. In terms of IUCN (2024) criteria, this entire area can be considered a single location/population because a fire event could potentially affect the complete top of the La Sepultura hill. This coastal mountain range is highly vulnerable to forest fires, with recurrent recorded events (e.g., 2011, 2016). Additional common activities of conservation concern include motorized sporting activities, cattle ranching, illegal logging of the *Nothofagus* stands (as denounced in 2023), and makeshift dumps on the top of the La Sepultura hill.

##### Additional specimens examined (paratypes).

**Chile** • **Región del Libertador General Bernardo O’Higgins**: Provincia de Cachapoal, Comuna de San Vicente de Tagua Tagua, sector Roblería del cerro La Sepultura, 1150 m a.s.l., 5 September 2022, *M.A. González & C. Soto s.n*. (EIF 17448, CONC, SGO).

##### Unvouchered observations.

**Chile. Región del Libertador General Bernardo O’Higgins**: Provincia de Colchagua, Comuna de Placilla, Cerro La Sepultura, roblería al suroeste de Cumbre, 1060 m a.s.l., 11 September 2022, observation by Christofer Olea (https://www.inaturalist.org/observations/307415343).

#### Gilliesia taguataguensis

Taxon classificationPlantaeEphemeropteraLeptophlebiidae

Espinoza & Nic.García sp. nov.

185E0A76-9211-50D9-83D2-91BFA42B5C77

urn:lsid:ipni.org:names:77375743-1

Figs 9, 10

##### Diagnosis.

*Gilliesia taguataguensis* differs from *Gilliesia isopetala* Ravenna by its flowers with two stamens and four staminodes (vs. three stamens and three staminodes), and all inner floral appendages reaching the tip of the staminal tube (vs. only frontal pair of inner floral appendages reaching the staminal tube, four lateral-dorsal less than half of the staminal tube’s length or lacking).

**Figure 9.**
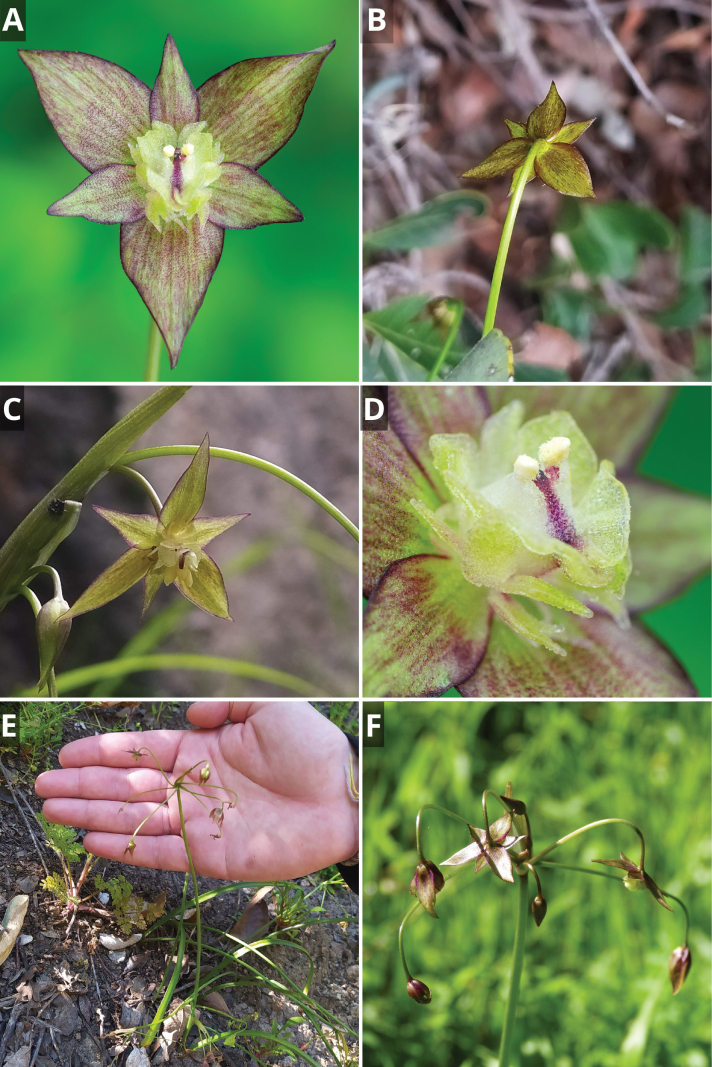
*Gilliesia taguataguensis* Espinoza & Nic. García. **A**. Frontal view of flower; **B**. Dorsal view of flower; **C**. Flower showing inverted position; **D**. Detail of staminal tube and floral appendages in lateral view; **E**. Habit; **F**. Inflorescence. Photos by Vicente Valdés (**A**, **D**), M. Matías Espinoza (**B**, **C**, **E**), and Nicolás García (**F**).

**Figure 10.**
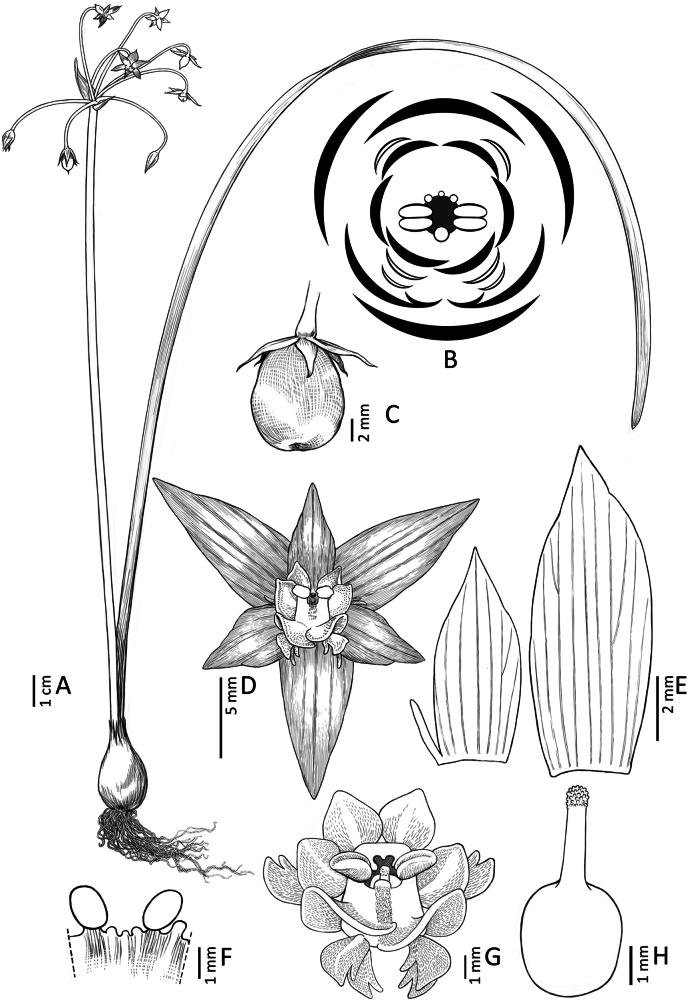
*Gilliesia taguataguensis* Espinoza & Nic. García. **A**. Habit; **B**. Floral diagram (large black crescents: tepals, inner lower pair showing tepaliferous appendages as smallest black crescents attached to tips; small white crescents: outer appendages; small black crescents: inner appendages; double ellipses: anthers; white circles: staminodes; dark gray circle: gynoecium); **C**. Fruit (lateral view); **D**. Flower (frontal view); **E**. Tepals (left: inner with tepaliferous appendage; right: outer); **F**. Staminal tube (open, internal view); **G**. Staminal tube and floral appendages; **H**. Gynoecium. Illustration by Daniel Martínez Piña.

##### Type.

Chile • Región del Libertador General Bernardo O’Higgins: Provincia de Cachapoal, Comuna de San Vicente de Tagua Tagua, El Naranjal, sector Poza Bruja, 306 m a.s.l., 27 August 2023, *N. García, C. Ulloa, M.M. Espinoza, C. Olea, J.A. Hernández & R.A. Alarcón 6809* (holotype: EIF 18606; isotypes: CONC, SGO).

##### Description.

Terrestrial herbs. **Bulbs** ovoid to narrowly ovoid, 27–30 × 9–15 mm, external cataphylls light brown, veins prominent. **Leaves** one, lorate, ~ 25–70 × 0.5–1.3 cm. **Scapes** one, cylindrical, hollow, 17–48 × 0.2–0.4 cm. **Spathe** 2-valvate, herbaceous, purplish to green, veins inconspicuous, free, **outer valve** lanceolate, 21–35 × 6–7 mm, veins 9–13, **inner valve** linear to lanceolate, 15–16 × 2–3 mm, veins four. **Inflorescence** a pseudo-umbel with 9–14 flowers; **pedicels** unequal, 3.0–6.7 cm long (with open flowers), usually with strongly recurved apical portions. **Tepals** six, free, membranous, vinaceous (purplish red) to greenish and tinged purplish, lanceolate to ovate, acute to acuminate, papillose on adaxial side, **outer** 8–10 × 3.8–4.5 mm, 5–11 veins, **inner** 4.5–8.0 × 2.5–3.0 mm, 1–3 veins. **Floral appendages** light green, sometimes with vinaceous tips and margins, mostly attached to the staminal tube, **outer (tepaliferous)** 2–4, awl-shaped to linear, attached to the base of inner tepals, 0.4–1.8 mm long, **middle** (staminal?) six, falcate to lanceolate, with apical half bent downwards, sometimes bifid, two lateral and two frontal pairs, 1.5–2.0 mm long, **inner (staminal)** six, flabellate to ovate, frontal pair with adjacent sides imbricate and apical half recurved 90°, 2.2–2.5 mm long. **Stamens** two, filaments triangular, 0.4 mm long, rarely three aborted anthers; **staminal tube** contiguous to filaments, greenish, sometimes with frontal vinaceous vein leading to frontal staminode, papillose, 2.0–3.0 mm long, **staminodes** four, triangular tips on apex of staminal tube, frontal solitary between functional stamens, 0.4 mm long, three towards back, median smaller (~ 0.1 mm) than lateral pair (~ 0.3 mm); **anthers** two, rarely three but smaller and sterile, yellow, dorsifixed near base, bent > 90° with apertures pointing outwards, 1.0–1.4 mm long. **Ovary** superior, spherical, 1.0–1.5 mm long, trilocular, 4–6 ovules per locule, biseriate; **style** straight to ascending, 0.9–1.7 mm long, reaching the anthers to slightly exerted in mature flowers; **stigma** capitate, terminal. **Capsules** spherical, 3-valved, 8 × 9 mm. **Seeds** not seen.

##### Distribution and habitat.

*Gilliesia taguataguensis* has been recorded in five locations in the eastern portion of the coastal mountain range that lies between the Cachapoal and Tinguiririca rivers (~ 34.5°S) at elevations between 300 and 960 m a.s.l. (Fig. 1A). In its type locality, it grows on a north- to northeast-facing slope in organic soils close to a creek and between rocks. The surrounding vegetation corresponds to a sclerophyllous arborescent scrub and forest, where the most abundant species are *Peumus boldus*, *Cryptocarya alba*, *Lithraea caustica* (Molina) Hook. & Arn., and *Retanilla trinervia* Hook. & Arn.

##### Phenology.

*Gilliesia taguataguensis* has been recorded in flowers from late August to early October. Mature fruits have not been seen, but it has been recorded fruiting from late September to October.

##### Etymology.

The specific epithet refers to the locality of Taguatagua, which currently corresponds to the municipality of San Vicente de Tagua Tagua, located to the southwest of the city of Rancagua in the O’Higgins region of Chile.

##### Vernacular name.

Although no popular common name is known for *Gilliesia taguataguensis*, we propose to name it “*brujita de Taguatagua*,” which means Taguatagua’s little witch, in reference to the name of the locality it was first noticed in (*Poza Bruja*, meaning Witch Pond).

##### Conservation status.

*Gilliesia taguataguensis* can be considered Endangered (EN) under criteria B1ab(iii), because its EOO is 15.2 km^2^ (< 5,000 km^2^) and it has been recorded in five locations. Its AOO has been estimated at 20 km^2^. The quality of its habitat is projected to decline given scenarios of climate change (Luebert and Pliscoff 2017, see P39 and P41). In addition, the area is at risk of forest fires and is subject to land use change for agricultural crops, motorized sporting activities, and goat and cattle ranching.

##### Additional specimens examined (paratypes).

**Chile** • **Región del Libertador General Bernardo O’Higgins**: Provincia de Cachapoal, Comuna de San Vicente de Tagua Tagua, El Naranjal, sector Poza Bruja, 490 m a.s.l., 29 August 2022, *M.M. Espinoza & J.A. Hernández 1* (EIF, CONC, SGO); • El Tambo, cerro Alto de Los Robles, 963 m a.s.l., 31 August 2024, *M.M. Espinoza 3* (EIF 18583); • Provincia de Colchagua, Comuna de San Fernando, Nincunlauta, sector Loma del Pangalillo, 395 m a.s.l., 08 September 2024, *M.M. Espinoza 4* (EIF 18584); • Quebrada Las Bandurrias, sector La Lechería, 400 m a.s.l., 31 August 2025, *J. Alarcón 1* (EIF 18630).

##### Unvouchered observations.

**Chile. Región del Libertador General Bernardo O’Higgins**: Provincia de Cachapoal, Comuna de San Vicente de Tagua Tagua, El Naranjal, cerros al oeste de Poza Bruja, 01 September 2023, observation by Christofer Olea (https://www.inaturalist.org/observations/307415344).

### Key to the species of Amaryllidaceae subf. Allioideae tribe Gilliesieae

[modified from Escobar (2012), Cádiz-Véliz (2021), García et al. (2022a), and Escobar et al. (2023)]

**Table:** 

1	Plants with 2–5 linear leaves and 1–3 scapes. Inflorescence 1–7-flowered. Androecium formed by six stamens, staminodes absent	**2**
–	Plants with 1–2 lanceolate to navicular leaves (3–4 in *Trichlora*) and 1 (–3) scape(s). Inflorescence 4–15-flowered. Androecium formed by 2–3 (–4) functional stamens and 1–3 staminodes	**13**
2	Perigone formed by three tepals with bristled margins. Outer filaments longer and alternating with inner filaments	***Schickendantziella trichosepala***
–	Perigone formed by six tepals with entire margins. All filaments of equal length	**3 *Miersia***
3	Flowers with two appendages above the staminal tube	**4**
–	Flowers with six appendages around the staminal tube or appendages absent	**5**
4	Floral appendages lorate to cuneiform, apex truncate, erose, and deflected, oriented frontward; white staminal tube featuring an elongated frontal lobe with a purple apical spot	***M. putaendensis***
–	Floral appendages oblong to subulate, apex entire, obtuse, and straight, oriented upward; bluish-green staminal tube with an erect, short, upper lobe without a purple spot	***M. leporina***
5	Tepals clearly reflexed on their distal half. Staminal tube with a globose deflected base. Floral appendages entire and filiform to narrowly lanceolate	***M. cornuta***
–	Tepals generally straight throughout or slightly reflexed. Staminal tube not globose at base. Floral appendages divided or absent	**6**
6	Tepals caudate over 2/3 of their length	**7**
–	Tepals acute to acuminate, rarely shortly (¹/5) caudate	**9**
7	Floral appendages absent. Filaments born apically, conspicuous and seeming a continuation of the tube, bent ~ 90° with stamens pointing outwards. Stigma trilobed	***M. humilis***
–	Floral appendages present. Filaments straight with stamens clumped around and close to the stigma. Stigma capitate	**8**
8	Staminal tube with a short apical reflexed rim. Filaments inconspicuous, inserted and born laterally on the inner face of the tube	***M. stellata***
–	Staminal tube conical with straight, lobed apex. Filaments conspicuous and a continuation of the staminal tube	***M. subandina* sp. nov**.
9	Tepals creamy white with two to three purple longitudinal stripes, rarely plain creamy white, perigone actinomorphic. Floral appendages absent or awl-shaped and shorter than 0.5 mm. Staminal tube conical, purplish; opening central and pointing towards the front of the flower	***M. raucoana***
–	Tepals plain light green to purplish or sometimes with a single central and broad purple longitudinal stripe (in *M. tenuiseta*), perigone zygomorphic. Floral appendages filiform or flat, bifid to trifid, longer than 0.5 mm. Staminal tube urceolate, whitish to greenish, or with a wide purple stripe on upper face; opening lateral, placed towards the lower side of the flower	**10**
10	Tepals acuminate to shortly caudate, apex straight or reflexed	**11**
–	Tepals acute, apex straight or inflexed	**12**
11	Flowers usually nodding, with upper tepals and stamens pointing downwards, upper, and lower tepals forming a right angle. Tepals 19–20 mm long, acuminate to shortly caudate	***M. nahuelbutensis* sp. nov**.
–	Flowers usually pointing to the front, upper and lower tepals wide open. Tepals 5–15 mm long, acute to acuminate	***M. chilensis***
12	Outer tepals lanceolate to linear-lanceolate. Appendages filiform, upper and lateral similar	***M. tenuiseta***
–	Outer tepals ovate to oval-lanceolate. Appendages flat, upper and lateral different	***M. minor***
13	Perigone formed by three tepals. Androecium formed by three stamens alternating with three cuneiform staminodes. Stigma trifid	**14 *Trichlora***
–	Perigone formed by 4–6 tepals. Androecium formed by 2–4 stamens and 1–4 deltoid staminodes. Stigma capitate (except *G. graminea*)	**15 *Gilliesia***
14	Tepals linear-lanceolate, 12–16 × 2–4 mm, generally yellow-greenish. Pedicels up to 4.5 cm long	***T. peruviana***
–	Tepals lanceolate to oval-lanceolate, 8–10 × 2–3 mm, generally purplish. Pedicels up to 8 cm long	***T. sandwithii***
15	Flowers slightly zygomorphic, perigone actinomorphic, tepals six. Floral appendages equal, nearly orbicular to semi-circular or cuneiform, entire, or appendages absent	**16**
–	Flowers strongly zygomorphic, perigone generally zygomorphic, tepals 4–6. Floral appendages unequal, inner frontal and larger, and several outer, shorter, entire to divided	**19**
16	Scapes 10–35 cm long, aerial portion usually shorter than 20 cm. Androecium formed by three stamens and 1–3 staminodes. Floral appendages absent	***G. miersioides* (= *Solaria miersioides* )**
–	Scapes up to 110 cm long, aerial portion taller than 20 cm. Androecium formed by 2–3 stamens and 0–4 staminodes. Floral appendages present	**17**
17	Flowers nodding, pointing downwards. Androecium formed by two stamens and 1–4 staminodes. Tepals green or green-yellowish, up to 4 cm long. Inner floral appendages cuneiform	***G. cuspidata* (= *Ancrumia cuspidata* )**
–	Flowers pointing to the front or upwards. Androecium formed by three stamens and 0–3 staminodes. Tepals vinaceous (purplish red), rarely green, up to 2.5 cm long. Inner floral appendages nearly orbicular, cordate to semi-circular	**18**
18	Tepals (9–)12–25 mm long, usually straight or reflexed at tips, sometimes inner tepals reflexed from base. Staminodes 1–3	***G. atropurpurea* (= *Gethyum atropurpureum* )**
–	Tepals 6–10 mm long, usually strongly reflexed from the base. Staminodes 0–1	***G. reflexa* sp. nov**.
19	Inner floral appendages flabellate to cuneiform, with crenulate to erose margins; outer appendages fimbriate	**20**
–	Inner floral appendages flabellate to flabellate-falcate, with entire margins; outer appendages entire or bifid	**21**
20	Perigone of four tepals forming a cross, rarely five, inner tepals shortly ovate, rarely lanceolate	***G. dimera***
–	Perigone of six tepals, inner tepals oval-lanceolate	***G. montana***
21	Perigone formed by six linear-lanceolate to ovate tepals. Inner floral appendages flabellate	**22**
–	Perigone formed by five (rarely six) lanceolate, oval-lanceolate or oval tepals. Inner floral appendages flabellate-falcate	**23**
22	Androecium formed by three stamens and three staminodes. Only frontal pair of inner floral appendages reaching the staminal tube, four lateral-dorsal inner appendages less than half of the staminal tube’s length or lacking	***G. isopetala***
–	Androecium formed by two stamens and four staminodes. All six inner floral appendages reaching the tip of the staminal tube	***G. taguataguensis* sp. nov**.
23	Flowers 1.0–2.5 cm long. Outer floral appendages filiform. Androecium usually with three staminodes. Stigma trilobed	***G. graminea***
–	Flowers 2.0–3.5 cm long. Outer floral appendages flat, falcate, and usually bifid. Androecium usually with (0–)2 staminodes. Stigma capitate	***G. scalae***

## Discussion

Overall, this study confirms previous phylogenetic hypotheses and the resolution of tribe Gilliesieae (Escobar et al. 2020; García et al. 2022a) but sheds further light on issues within the *Gilliesia* s.l. clade. Regarding *Miersia*, both new species are positioned within the *Miersia* II clade (sensu García et al. 2022a; Fig. 2), which remains highly unresolved given the molecular markers currently used in this group (Escobar et al. 2020). However, it is interesting to note that *Miersia subandina* is retrieved with low support (BS = 68) as forming a clade with *M. humilis* and *M. stellata*, with which it shares the presence of caudate tepals. With the two novelties presented here, *Miersia* is composed of 11 species, all endemic to Chile, and extends its southern range to Malleco Province in the La Araucanía Region (Fig. 1) due to the description of *M. nahuelbutensis*.

On the other hand, the phylogenetic analysis presented here provides further support to circumscribe *Gilliesia* in a wide sense, including *Solaria*, *Gethyum*, and *Ancrumia*. The paraphyly of *Gilliesia*—given the sister relationships of *Solaria miersioides* Phil. (treated here as *Gilliesia miersioides* (Phil.) M.F.Fay & Christenh.) with *Gilliesia graminea* Lindl. (type species of *Gilliesia*) and of *Gethyum atropurpureum* Phil. (treated here as *Gilliesia atropurpurea* (Phil.) M.F.Fay & Christenh.) with *Gilliesia isopetala* Ravenna—has been noted since the work by Escobar (2012; also in Escobar et al. 2020). Regarding the second case, our phylogenetic results confirm the close relationship between *G. atropurpurea* and *G. isopetala* and place them in a larger clade (BS = 97) that includes the newly described *G. reflexa* and *G. taguataguensis* (Fig. 2). *Gilliesia reflexa* is morphologically similar to *G. atropurpurea*, with which it shares its rounded inner floral appendages and star-shaped perigone, but in our phylogenetic results it is retrieved with low support (BS = 76) as sister to *G. taguataguensis* (Fig. 2), which in turn is most similar to *G. isopetala* (see key to Gilliesieae species). With the exception of *G. atropurpurea*, which has a wider distribution (Escobar et al. 2023), this clade is centered in the Cachapoal River basin, around which the remaining three species are narrow endemics within coastal mountain ranges (Fig. 1). Interestingly, both *G. reflexa* and *G. taguataguensis* are restricted to the Coastal mountain range that lies between the Cachapoal and Tinguiririca River basins, which is a poorly studied area in terms of its flora. With the description of these two narrowly endemic and therefore threatened species, this particular mountainous complex gains value as a priority site for the conservation of Gilliesieae diversity.

Regarding the species formerly known as *Ancrumia cuspidata* Harv. ex Baker, here treated as *Gilliesia cuspidata* (Harv. ex Baker) M.F.Fay & Christenh., our phylogenetic analysis shows low support for the sister relationship of this species with the rest of the *Gilliesia* clade and strong support for that clade (Fig. 2; Escobar et al. 2020), in contrast to a previous concatenated analysis that suggested low support for a *Gilliesia* clade including *Ancrumia* (García et al. 2022a). A generic circumscription of *Gilliesia*, including *Ancrumia*, *Gethyum*, and *Solaria*, renders this genus monophyletic according to the currently available phylogenetic hypothesis of Gilliesieae. In this wider sense, *Gilliesia* is composed of 10 species and is diagnosed in the context of Gilliesieae taxonomy by its perigone formed by 4–6 tepals and its androecium formed by 2–3 (rarely 4) stamens and 1–4 deltoid staminodes.

A comprehensive evolutionary study and generic taxonomy of tribe Gilliesieae is still pending due to the same issues mentioned in García et al. (2022a), including the lack of DNA sequences from *Trichlora* and *Schickendantziella* and the desirability for data from multiple low-copy nuclear genes to resolve evolutionary relationships.

## Supplementary Material

XML Treatment for Miersia nahuelbutensis

XML Treatment for Miersia subandina

XML Treatment for Gilliesia reflexa

XML Treatment for Gilliesia taguataguensis

## Additional information

### Use of AI

No use of AI was reported.

### Author ORCIDs

Nicolás García https://orcid.org/0000-0001-9003-1510

Paula Zúñiga-Acevedo https://orcid.org/0009-0005-2714-720X

Matías A. González https://orcid.org/0009-0008-4830-2899

M. Matías Espinoza https://orcid.org/0009-0009-6536-8950

Constanza Soto https://orcid.org/0009-0002-2847-6206

### Data availability

All of the data that support the findings of this study are available in the main text and in Zenodo (https://doi.org/10.5281/zenodo.17372175).
